# Achados à RM dinâmica e a escala mJOA: Estabelecimento de correlações na avaliação clínica

**DOI:** 10.1055/s-0045-1811929

**Published:** 2025-11-18

**Authors:** Ricardo André Acácio dos Santos, Raphael de Rezende Pratali, Mariana Demétrio de Sousa Pontes, Carlos Fernando P. S. Herrero

**Affiliations:** 1 Departamento de Ortopedia e Anestesiologia, Faculdade de Medicina de Ribeirão Preto, Universidade de São Paulo, Ribeirão Preto, SP, Brasil; 2 Departamento de Ortopedia, Hospital do Servidor Público Estadual, São Paulo, SP, Brasil

**Keywords:** diagnóstico, ressonância magnética, vértebras cervicais, cervical vertebrae, diagnosis, magnetic resonance

## Abstract

**Objetivo:**

Investigar a correlação entre a pontuação na escala modificada da Japanese Orthopaedic Association (mJOA) e os achados de ressonância magnética dinâmica (RMD) em pacientes diagnosticados com mielopatia cervical degenerativa (MCD).

**Métodos:**

Conduzimos um estudo de coorte retrospectiva. Todos os pacientes foram submetidos a um exame de RMD da coluna cervical usando o mesmo equipamento. Os parâmetros anatômicos avaliados foram o diâmetro da medula espinhal (DME) e a largura do canal vertebral (LCV). O DME foi medido como a distância entre o ponto médio da porção posterior do disco intervertebral e a margem anterior do ligamento amarelo. A LCV foi medida como a distância entre as margens anterior e posterior da medula espinhal no ponto exato de avaliação do DME. A escala mJOA foi escolhida para avaliação do estado funcional. As confiabilidades intra e interobservador dos parâmetros morfométricos da ressonância magnética (RM) foram calculadas segundo o coeficiente de correlação intraclasse (CCI), e valores de *p* < 0,05 foram considerados estatisticamente significativos.

**Resultados:**

A avaliação intraobservador do DME teve concordância quase perfeita, e a avaliação interobservador da LCV apresentou forte concordância. A pontuação na mJOA variou de 6 a 18. Houve uma correlação fraca e não estatisticamente significativa entre os parâmetros DME e LCV.

**Conclusão:**

Não identificamos uma correlação entre as medidas do canal vertebral cervical obtidas em exames de RMD e a gravidade clínica dos pacientes com MCD medida pela escala mJOA. Devido ao pequeno tamanho da amostra, esses achados devem ser interpretados com cautela.

## Abstract (en)

**Objective:**

To investigate the correlation between the score on the modified Japanese Orthopaedic Association (mJOA) scale and the dynamic magnetic resonance (DMR) findings in patients diagnosed with degenerative cervical myelopathy (DCM).

**Methods:**

We conducted a retrospective cohort study. All patients underwent a DMR examination of the cervical spine using the same device. The anatomic parameters evaluated were the spinal canal diameter (SCD) and the spinal canal width (SCW). The SCD was measured as the distance between the midpoint of the posterior portion of the intervertebral disc and the anterior margin of the yellow ligament. The SCW was measured as the distance between the anterior and posterior margins of the spinal cord at the exact point used to assess the SCD. The mJOA scale was chosen to assess the functional status. The intra and interobserver reliability of the morphometric parameters of the magnetic resonance imaging (MRI) were calculated using the intraclass correlation coefficient (ICC), and values of *p* < 0.05 were considered statistically significant.

**Results:**

Regarding the intraobserver evaluation of the SCD, an almost perfect agreement was achieved, and the SCW's interobserver evaluation presented a strong agreement. The mJOA score ranged from 6 to 18, and there was a weak, non-statistically significant correlation between the SCD and SCW parameters.

**Conclusion:**

We could not identify a correlation involving the cervical spinal canal measurements obtained from DMR exams and the clinical severity of patients with DCM measured using the mJOA score. Due to the small sample size, these findings should be interpreted with caution.

## Introdução

A mielopatia cervical degenerativa (MCD) é a causa mais comum de disfunção da medula espinhal em pacientes com mais de 60 anos. 1 O desenvolvimento de MCD está associado à degeneração da coluna cervical relacionada à idade e à compressão da medula espinhal pelo tecido degenerado anormal. 1 2 Pacientes com MCD sintomática podem apresentar uma combinação de disfunções motora, sensorial e autonômica e, consequentemente, redução da qualidade de vida. 1 2

Diversos questionários podem ser usados para avaliar a gravidade da deficiência física, o estado clínico do paciente e a eficácia do tratamento. 3 Entre esses instrumentos, a pontuação na escala modificada da Japanese Orthopaedic Association (mJOA) tornou-se uma das medidas de desfecho mais utilizadas para a avaliação do estado funcional em pacientes com MCD. 4 5 6

Embora a avaliação da compressão da medula espinhal e o diagnóstico de MCD sejam baseados principalmente na avaliação clínica, o papel crucial da ressonância magnética (RM) neste processo é bem reconhecido. 7 Embora estudos anteriores 8 9 com achados de RM tenham demonstrado que esse exame auxilia na determinação do prognóstico do tratamento cirúrgico, nem todos os parâmetros foram úteis. Os fatores que demonstraram ter influência na avaliação incluem a duração da hiperintensidade em T2, a estenose congênita, a espondilolistese cervical e a hipointensidade em T1. 8 9 10 11 12 13 14

A RM dinâmica (RMD), que inclui a avaliação da coluna cervical durante a flexão e a extensão, pode refletir melhor o impacto biomecânico em tempo real do movimento na medula espinhal do que a RM estática. 3 8 9 Isto é particularmente relevante na MCD, em que os sintomas muitas vezes flutuam conforme a posição do pescoço. Além disso, a compressão transitória da medula espinhal pode não ser totalmente capturada em imagens convencionais. 3 8 9 Recentemente, outros estudos 3 8 9 investigaram o papel da RM em pacientes com MCD, e demonstraram alterações significativas nas medidas do canal vertebral cervical em um subgrupo de indivíduos acometidos; os autores afirmaram que esses resultados podem explicar os achados do exame clínico e, consequentemente, influenciar os desfechos do tratamento cirúrgico.

Portanto, estudamos dados de um subconjunto de pacientes com MCD de uma pesquisa prospectiva para avaliar a correlação entre os achados clínicos (ou seja, a pontuação na mJOA) e os achados de RMD.

## Materiais e Métodos

### Delineamento Experimental e Coleta de Dados

O Comitê de Ética e o Conselho de Revisão Interna do hospital aprovaram o estudo, e todos os participantes assinaram o termo de consentimento livre e esclarecido antes do início da coleta de dados. Esta pesquisa foi definida como um estudo de coorte prospectiva com o objetivo de avaliar a confiabilidade de uma técnica de imagem por RMD cervical em pacientes diagnosticados com MCD com base em achados preliminares já publicados.

Os participantes tinham diagnóstico clínico de MCD confirmado por RM e, posteriormente, foram submetidos a procedimentos cirúrgicos padrão para o tratamento. Os critérios de inclusão foram o preenchimento do questionário mJOA, a realização do exame de RMD e o consentimento para a participação no estudo. Os pacientes que haviam sido submetidos a cirurgias prévias da coluna cervical ou que apresentavam outras doenças ortopédicas, neurológicas ou psiquiátricas que pudessem influenciar os desfechos clínicos foram excluídos. Dos 168 candidatos que responderam ao questionário mJOA, 18 pacientes (14 homens e 4 mulheres) preencheram os critérios de inclusão e tinham imagens de RMD disponíveis para análise subsequente.

### Aquisição e Avaliação de Imagens

Todos os pacientes incluídos foram submetidos a uma avaliação por RMD da coluna cervical em equipamento Achieva padronizado de 1,5 Tesla fabricado pela Philips. A análise das imagens foi realizada com o programa OsiriX MD, versão 7.0, de 64 bits (Primeo SARL), em aumento de 300%. As imagens foram analisadas por dois cirurgiões de coluna independentes. Um desses observadores repetiu a avaliação após 30 dias para analisar a confiabilidade intraobservador, usando o mesmo protocolo, computador e programa.

### Medidas Anatômicas

**Fig. 1:**
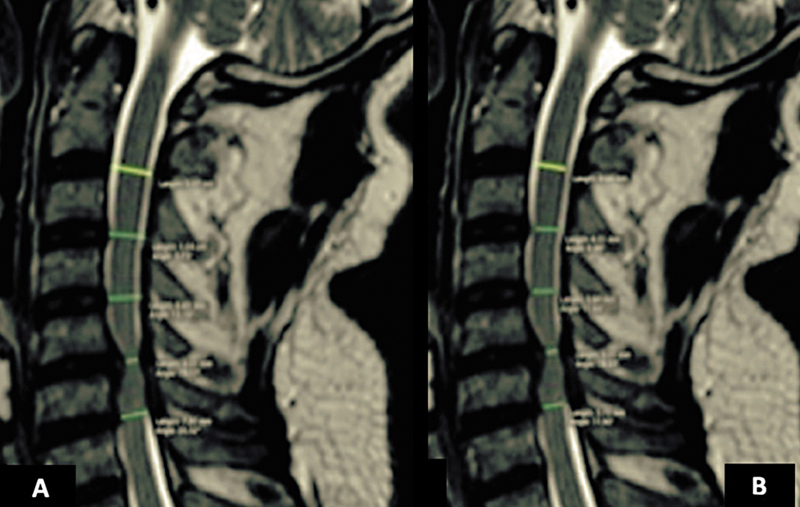
Imagem de ressonância magnética sagital cervical ponderada em T2. ( **A** ) Largura do canal vertebral. ( **B** ) Diâmetro da medula espinhal.

Os parâmetros anatômicos avaliados foram o diâmetro da medula espinhal (DME) e a largura do canal vertebral (LCV). A LCV foi calculada medindo-se a distância entre o ponto médio da parte posterior do disco intervertebral e o limite anterior do ligamento amarelo ( Fig. 1A ). O DME foi determinado como a distância da margem anterior à margem posterior da medula espinhal no mesmo ponto de medida da LCV ( Fig. 1B ). Todas as medidas foram lineares e registradas em milímetros (±1,0 mm), capturadas em imagens da linha média, em sequências ponderadas em T2 no plano sagital, nas posições neutra, em flexão e em extensão, nos espaços discais C2-C3 a C6-C7.

### Avaliação Clínica

A escala mJOA foi escolhida para a avaliação do estado funcional. Esta escala de 18 pontos sobre a MCD quantifica separadamente a função motora dos membros superiores (5 pontos) e inferiores (7 pontos), a sensibilidade (3 pontos) e o controle de esfíncteres (3 pontos). 5

### Análise Estatística

Para a análise estatística, foi usado o programa STATA13 (StataCorp LLC). As confiabilidades intra e interobservador dos parâmetros morfométricos da RMD foram calculadas usando o coeficiente de correlação intraclasse (CCI), com um IC95%. Valores de CCI de 0,00 a 0,20 indicaram concordância baixa, de 0,21 a 0,40, concordância razoável, de 0,41 a 0,60, concordância moderada, de 0,61 a 0,80, concordância forte, e de 0,81 a 1,00, concordância quase perfeita. A análise de correlação estatística considerou a gravidade da MCD, representada pela pontuação na mJOA, e as medidas dinâmicas usando o teste de correlação de postos de Spearman. Valores de *p* < 0,05 foram considerados estatisticamente significativos.

## Resultados

### Pacientes

Ao todo, 18 pacientes elegíveis concluíram o protocolo de RMD, preencheram os critérios de inclusão e foram analisados neste estudo. Os pacientes eram 14 homens e 4 mulheres, com média de idade de 60 (variação: 37–76) anos.

### Confiabilidade Interobservador

Na avaliação do DME, os valores médios do CCI de todos os níveis discais na confiabilidade interobservador foram de 0,90 em posição neutra e de 0,92 em flexão e extensão ( Tabela 1 ). A confiabilidade interobservador da LCV apresentou um valor médio de CCI de 0,80, 0,88 e 0,87 nas posições neutra, em flexão e em extensão, respectivamente ( Tabela 1 ).

**Tabela 1:** Confiabilidade interobservador quanto à largura do canal espinhal e ao diâmetro da medula espinhal segundo o coeficiente de correlação intraclasse

	Flexão (IC95%)	Posição neutra (IC95%)	Extensão (IC95%)
	**Largura do canal espinhal**		
**C2** – **C3**	0,94 (0,86–0,98)	0,95 (0,87–0,98)	0,97 (0,92–0,98)
**C3** – **C4**	0,89 (0,73–0,96)	0,98 (0,95–0,99)	0,95 (0,88–0,98)
**C4** – **C5**	0,87 (0,66–0,95)	0,94 (0,86–0,98)	0,89 (0,70–0,95)
**C5** – **C6**	0,93 (0,83–0,97)	0,93 (0,81–0,97)	0,92 (0,78–0,98)
**C6** – **C7**	0,90 (0,75–0,96)	0,83 (0,56–0,93)	0,91 (0,78–0,96)
	**Diâmetro da medula espinhal**		
**C2** – **C3**	0,84 (0,59–0,94)	0,96 (0,91–0,98)	0,94 (0,86–0,98)
**C3** – **C4**	0,92 (0,80–0,97)	0,95 (0,88–0,98)	0,94 (0,84–0,97)
**C4** – **C5**	0,84 (0,57–0,94)	0,91 (077,–0,96)	0,95 (0,89–0,98)
**C5** – **C6**	0,95 (0,87–0,98)	0,95 (0,87–0,98)	0,90 (0,75–0,96)
**C6** – **C7**	0,73 (0,29–0,90)	0,82 (0,54–0,93)	0,82 (0,52–0,93)

### Confiabilidade Intraobservador

Em relação à confiabilidade intraobservador dos valores de DME, o CCI médio foi de 0,97 em posição neutra, de 0,96 em flexão e de 0,97 em extensão; todos os níveis discais apresentaram CCI superior a 0,90 em todas as posições. Na avaliação da LCV, os valores médios do CCI foram de 0,94 em posição neutra, de 0,87 em flexão e de 0,94 em extensão. Os valores de CCI em todos os níveis discais foram superiores a 0,8 em todas as posições. A Tabela 2 apresenta a descrição completa dos valores do CCI em cada posição e nível discal.

**Tabela 2:** Confiabilidade intraobservador quanto à largura do canal espinhal e ao diâmetro da medula espinhal segundo o coeficiente de correlação intraclasse

	Flexão (IC95%)	Posição neutra (IC95%)	Extensão (IC95%)
	**Largura do canal espinhal**		
**C2** – **C3**	0,96 (0,91–0,98)	0,98 (0,97–0,99)	0,99 (0,97–0,99)
**C3** – **C4**	0,98 (0,95–0,99)	0,99 (0,98–0,99)	0,98 (0,97–0,99)
**C4** – **C5**	0,94 (0,86–0,98)	0,96 (0,91–0,98)	0,99 (0,99–0,99)
**C5** – **C6**	0,98 (0,96–0,99)	0,98 (0,94–0,99)	0,93 (0,83–0,97)
	**Diâmetro da medula espinhal**		
**C2** – **C3**	0,84 (0,59–0,94)	0,96 (0,91–0,98)	0,94 (0,86–0,98)
**C3** – **C4**	0,92 (0,80–0,97)	0,95 (0,88–0,98)	0,94 (0,84–0,97)
**C4** – **C5**	0,84 (0,57–0,94)	0,91 (0,77–0,96)	0,95 (0,89–0,98)
**C5** – **C6**	0,95 (0,87–0,98)	0,95 (0,87–0,98)	0,90 (0,75–0,96)

### Correlação entre os Achados à RMD e a Pontuação na mJOA

**Fig. 2:**
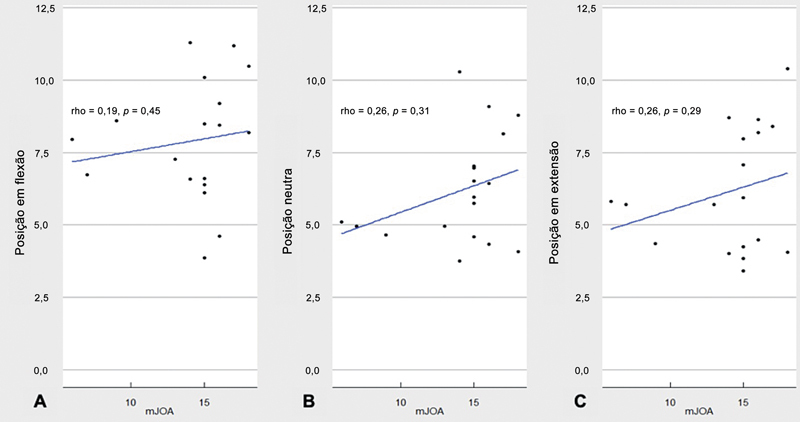
Correlação entre a pontuação na escala modificada da Japanese Orthopaedic Association (mJOA) e o diâmetro da medula espinhal (DME) nas posições em flexão ( **A** ), neutra ( **B** ) e em extensão ( **C** ).

A pontuação na mJOA variou de 6 a 18, com mediana de 15. O teste de classificação de Spearman, utilizado para avaliar a correlação entre os resultados obtidos na avaliação da pontuação na mJOA e o DME, demonstrou uma correlação positiva baixa na posição em flexão (0,191), com *p* = 0,448 ( Fig. 2A ), uma relação monotônica positiva e baixa em posição neutra (0,255), com *p* = 0,307 ( Fig. 2B ), e uma correlação positiva baixa na posição em extensão (0,265), com *p* = 0,288 ( Fig. 2C ).

**Fig. 3:**
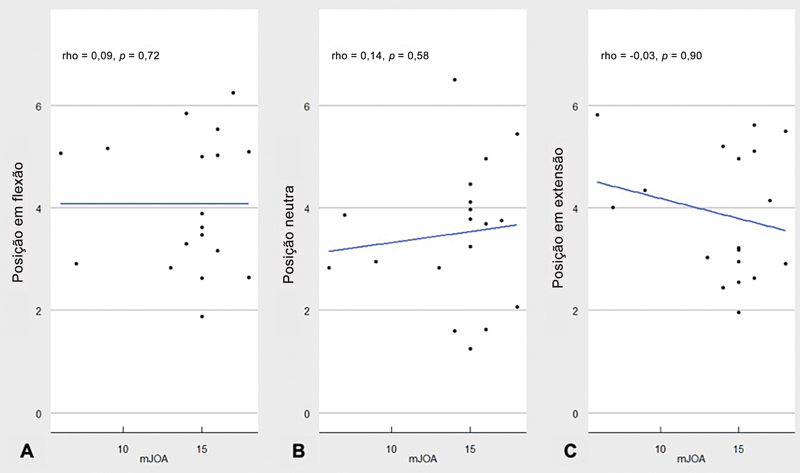
Correlação entre a pontuação na escala modificada da Japanese Orthopaedic Association (mJOA) e a largura do canal vertebral (LCV) nas posições em flexão ( **A** ), neutra ( **B** ) e em extensão ( **C** ).

Ao avaliar a correlação entre a pontuação na mJOA e a LCV, o teste de classificação de Spearman evidenciou uma correlação positiva muito baixa (0,089), com *p* = 0,724, na posição em flexão ( Fig. 3A ), uma correlação positiva baixa (0,14), com *p* = 0,507, em posição neutra ( Fig. 3B ), e uma correlação negativa baixa (−0,032), com *p* = 0,898, na posição em extensão ( Fig. 3C ).

## Discussão

Este estudo não revelou uma associação clara entre os achados de RMD e as pontuações na escala mJOA em pacientes com MCD. A MCD é a causa mais comum de disfunção da medula espinhal, e é caracterizada pela redução das medidas do canal vertebral por alterações degenerativas exacerbadas pelos movimentos da coluna cervical que podem ser observadas à RMD cervical. 15 16 Embora a literatura 3 4 5 6 7 8 9 10 11 12 13 14 15 16 17 apresente visões divergentes sobre a relação entre os achados de RM e as características clínicas da MCD, este estudo é, pelo que sabemos, o primeiro a medir parâmetros morfométricos do canal vertebral cervical em exames de RMD e correlacioná-los à pontuação na escala mJOA.

A RM é considerada uma importante ferramenta prognóstica em pacientes com MCD, devido à publicação de evidências que apoiam esse conceito e indicam que a redução na área transversal do canal espinhal cervical e as alterações de sinal na medula espinhal, nestes indivíduos, estão significativamente associadas à gravidade neurológica, à prevalência de manifestações clínicas específicas e à possibilidade de recuperação neurológica. 18 19 20 Em pacientes com MCD, a manifestação clínica é decorrente de alterações anatômicas degenerativas que causam compressão do canal vertebral. A RM da coluna cervical fornece imagens estáticas da compressão da medula espinhal. 17 18 19 No entanto, está bem demonstrado 15 que a compressão dinâmica resultante da hipermobilidade e da instabilidade da articulação cervical pode agravar um estreitamento existente do canal vertebral. Assim, os dados de RMD demonstraram que as diferentes posições (neutra, em flexão e em extensão) influenciam as medidas do diâmetro do canal vertebral cervical e da medula espinhal. 9

Esta pesquisa traz dados sobre os parâmetros morfométricos do canal vertebral e da medula espinhal cervical em pacientes com MCD com base em exames de RM adquiridos nas posições neutra, em flexão e em extensão. Avaliamos as confiabilidades inter e intraobservador dos parâmetros morfométricos da coluna cervical adquiridos com base no protocolo de RMD adotado em nossa instituição. 21 A medida de todos os parâmetros dinâmicos apresentou pelo menos concordância substancial quanto às confiabilidades inter e intraobservador. Da mesma forma, Yu et al. 22 relataram que a RMD é um método adequado para a identificação da instabilidade da coluna cervical. Outros autores 15 mostraram que pacientes com instabilidade da coluna cervical apresentaram pontuações piores na mJOA e na classificação de Nurick e sinais eletrofisiológicos piores do que os dos pacientes sem instabilidade. Além disso, Nigro et al. 16 sugeriram que os cirurgiões podem identificar mais achados associados à MCD e ao agravamento da compressão com as imagens de RMD do que com a RM cervical tradicional.

A escala mJOA, uma ferramenta útil na avaliação do MCD, foi analisada quanto à confiabilidade e validade. 5 6 Não existe um critério clínico ideal para avaliar pacientes com sintomas de compressão medular. A escala mJOA foi escolhida por ser o critério mais utilizado em estudos clínicos e por ser de fácil aplicação. Portanto, como a correlação entre os achados da RMD cervical e a gravidade da doença permanece controversa, este estudo buscou estabelecer uma correlação entre os achados da RMD cervical e a pontuação na escala mJOA. 5 6 No entanto, a correlação entre a pontuação na escala mJOA e os parâmetros morfométricos dinâmicos da coluna cervical não foi estatisticamente significativa. Além disso, embora a correlação não tenha atingido um nível de significância, o que pode ter ocorrido devido a um problema de tamanho de amostra, nenhuma das posições cervicais apresentou correlações maiores do que as demais. Isso pode sugerir que não há uma linha reta clara entre o diâmetro do canal vertebral e a mielopatia, mas que o fator mais significativo na predição de mudanças nas condições clínicas pode ser as mudanças na intensidade do sinal da medula espinhal, como visto na RM com tensor de difusão ( *diffusion tensor imaging* , DTI, em inglês). 23 A ausência de correlação positiva entre a pontuação na mJOA e a LCV na posição em extensão também pode ser explicada pelo fato de o grupo de pacientes estudados apresentar níveis iniciais e de mJOA elevados.

Diversas limitações devem ser consideradas na interpretação de nossos achados. O tamanho relativamente pequeno da amostra e a homogeneidade na gravidade dos achados clínicos, representada pela variação limitada nas pontuações na mJOA, podem ter introduzido um viés de amostragem e influenciado nossos resultados. Uma coorte maior de pacientes, com uma gama mais ampla de pontuações na mJOA, pode revelar correlações estatisticamente significativas entre os achados da RMD cervical e os desfechos clínicos. Além disso, este estudo não incluiu uma avaliação das alterações do sinal da RM da medula espinhal, que podem servir como indicadores de achados clínicos mais graves e, consequentemente, piores pontuações na mJOA. Ademais, a exclusão de anomalias do sinal da medula espinhal—como hiperintensidade em T2 e métricas baseadas em DTI—representa uma limitação significativa desta análise, visto que essas variáveis podem capturar melhor o comprometimento neural subjacente e explicar déficits funcionais com mais precisão do que as medidas morfométricas isoladamente. Do ponto de vista clínico, nossos achados sugerem que, sozinhas, as medidas morfométricas da RMD podem não ser suficientes para estimar o comprometimento funcional em pacientes com MCD, o que destaca a necessidade de uma abordagem de imagem mais abrangente que inclua a avaliação do sinal da medula espinhal. Essa percepção reforça a importância da avaliação multimodal no diagnóstico e no prognóstico da MCD. Apesar dessas limitações, este estudo apresenta uma maneira inovadora de correlacionar os achados à RMD e à pontuação na mJOA, e representa um modelo para estudos futuros com populações de pacientes maiores e mais heterogêneas, bem como com a inclusão de métricas de intensidade do sinal da medula espinhal, para elucidar melhor a complexa relação entre a morfologia dinâmica do canal espinhal e a apresentação clínica na MCD.

## Conclusão

Nossos resultados são conflitantes em relação a estudos já publicados, pois não foi possível identificar uma correlação entre as medidas do canal vertebral cervical à RMD e a gravidade clínica da MCD de acordo com a escala mJOA. Assim, nossos resultados reforçam a necessidade de mais pesquisas sobre RMD, uma vez que os movimentos inerentes da coluna cervical estão associados à fisiopatologia da MCD.
